# Supplementary material for: Content-rich biological network constructed by mining PubMed abstracts
Source: BMC Bioinformatics. 2004 Oct 8;5:147. doi: 10.1186/1471-2105-5-147 (PMC528731; doi:10.1186/1471-2105-5-147)
Supplement: Additional File 2 — The original results of the above study (non-essential files are deleted to keep the file size under the limit set by BMC bioinformatics). [file 1471-2105-5-147-S2.bz2 › chilibotAdditionalFile2/dip20/274ID1922387E1094/html/MAPK9_JUN.html]

 


 **MAPK9** and **JUN** 
  
Found 316 abstracts in PubMed, retrieved 20.  
 

 What does Google say? 
 PDF only 
| .edu only 

---

**Interactive relationship** (e.g. stimulation, inhibition, etc)

**Stimulatory relationship**- JNK2  [ **MAPK9** ]  transcript inhibition abrogated shear induced COX 2, EP2 and EP3a1 mRNA upregulation, as well as c  **jun**  phosphorylation.  Ref: 12743126 J Biol Chem< MedlineTA>J Biol Chem,
**Neutral relationship**

**Non-interactive relationship** (e.g. studied together, co-existance, homology, etc.)

- Exposure of T C 28a2 chondrocytic cells to fluid shear results in co regulation of c  **jun**  N terminal kinase2  [ **MAPK9** ]  JNK2  [ **MAPK9** ] , c  **jun**  and COX 2 as well as concomitant downstream expression of prostaglandin receptors EP2 and EP3a1.  Ref: 12743126 J Biol Chem< MedlineTA>J Biol Chem,
- These same peptides also inhibited JNK2  [ **MAPK9** ]  mediated phosphorylation of c  **Jun**  and ATF2, suggesting that transcription factors, MKK4, and the JIP scaffold compete for docking to JNK.  Ref: 12788955 J Biol Chem< MedlineTA>J Biol Chem,
- Thus, we suspected that either  **Jun**  N terminal kinase 2 JNK 2  [ **MAPK9** ]  or Src family proteins were involved in HLA DR mediated signaling.  Ref: 12842758 Cytokine, 2003
- The reduction appeared to be due to relocalization of TNFRI from the cell surface and was reflected in the elimination of TNF alpha induced  **Jun**  kinase  [ **MAPK9** ]  activity.  Ref: 12768019 J Virol, 2003
- Hypertonicity induced by NaCl, but not by urea or mannitol, up regulates expression of the gamma subunit of Na K ATPase in cells of the murine inner medullary collecting duct line IMCD3 by activation of the  **Jun**  kinase  [ **MAPK9** ]  2 JNK2  [ **MAPK9** ]  pathways.  Ref: 12746499 Proc Natl Acad Sci U S A< MedlineTA>Proc Natl Acad Sci U S A, 2003
- The activation of one of these, the  **Jun**  kinase  [ **MAPK9** ]  pathway, has been implicated in apoptotic responses to DNA damage, cell stress and cytotoxic drugs.  Ref: 12860462 Drug Resist Updat, 2003
- Shear induced cyclooxygenase 2 via a JNK2  [ **MAPK9** ]  c  **jun**  dependent pathway regulates prostaglandin receptor expression in chondrocytic cells.  Ref: 12743126 J Biol Chem< MedlineTA>J Biol Chem, 2003
- Hence, a biochemical pathway exists wherein fluid shear activates COX 2, via a JNK2  [ **MAPK9** ]  c  **jun**  dependent pathway, which in turn elicits downstream EP2 and EP3a1 mRNA synthesis.  Ref: 12743126 J Biol Chem< MedlineTA>J Biol Chem, 2003
- Functional knock out experiments using an antisense c  **jun**  oligonucleotide revealed the abolition of shear induced COX 2, EP2 and EP3a1, but not JNK2  [ **MAPK9** ] , transcripts.  Ref: 12743126 J Biol Chem< MedlineTA>J Biol Chem, 2003
- By immunoblotting, these proteins were identified as c  **Jun**  N terminal kinase JNK 2  [ **MAPK9** ]  and p38 mitogen activated protein kinase MAPK delta, respectively, both of which are believed to be involved in apoptosis induction by CDDP.  Ref: 12824923 Cancer Sci< MedlineTA>Cancer Sci, 2003
- 5 dihydroxyvitamin D3 inhibits ultraviolet B induced apoptosis,  **Jun**  kinase  [ **MAPK9** ]  activation, and interleukin 6 production in primary human keratinocytes.  Ref: 12858333 J Cell BiochemJ Cell Biochem, 2003
- Role of  **Jun**  and  **Jun**  kinase  [ **MAPK9** ]  in resistance of cancer cells to therapy.  Ref: 12860462 Drug Resist Updat, 2003
- 3 Through activation of the  **Jun**  kinase  [ **MAPK9** ]  pathway AP 1  [ **JUN** ]  is induced, leading to cell cycle arrest in the G0 G1 phase.  Ref: 12790790 Endocr Relat Cancer, 2003
- We have also found that IFNalpha induced apoptosis depends upon activation of the NH 2 terminal  **Jun**  kinase  [ **MAPK9** ]  1 Jnk 1 and p 38 mitogen activated protein kinase,.  Ref: 12700650 Cell Death Differ, 2003
